# Schizophrenia, a Narrow Scope of Negative Symptoms in Correlation with Multiple Factors

**DOI:** 10.1192/j.eurpsy.2026.12203

**Published:** 2026-07-31

**Authors:** S. F. Abu Dayeh, Q. H. Al-Rimawi

**Affiliations:** Psychiatry, MOH, Amman, Jordan

## Abstract

**Introduction:**

This study is conducted to observe negative symptoms details in correlation with multiple factors. 90 patients(n=90) diagnosed with schizophrenia were interviewed and observed in the outpatient clinic during there follow-up appointments.

**Objectives:**

- **First question:** Are there any correlations between severity of negative symptoms and factors such as age, duration and educational level ?
- **Second question:** What is the relationship between compliance to medications and severity of negative symptoms?
- **Third question:** What are the differences between the specific negative symptoms developed between male and female patients as measured by PANSS?

**Methods:**

90 patients with schizophrenia were observed and interviewed in the outpatient clinic during their follow up appointments, and patients’ informed consent was obtained. Pearson and Spearman’s Rho correlation tests, Kruska-Wallis test, independent t-test and PANSS, (Positive and Negative Syndrome Scale of Schizophrenia), were used for this study.

**Results:**

**Table 1.** Pearson and Spearman’s Rho correlation tests results between age, and duration of the illness, and educational level with the negative symptoms Table 1. Long description.

Variables		Age	Duration of the illness	Educational level
Negative symptoms	Correlation Coefficient	.303**	0.324**	–0.581**
	Sig. (2-tailed)	0.005	0.002	0.000

**Table 2.** Kruska-Wallis test for the differences in compliance to medications and the severity of negative symptoms Table 2. Long description.

Compliance to medications	N	Mean Rank	Kruska-Wallis H	Sig.
poor	11	63.50	16.875	0.000
moderate	11	47.95		
good	59	36.12		
forced	3	71.00		

Gender	N.	Mean	Std. Deviation	T	Df	Sig.
Male	49	26.65	7.551	1.681	88	0.096
**Female**	**41**	**23.93**	**7.792**

**Conclusions:**

Table 1 analysis: age and duration of the illness are positively correlated to negative symptoms. Where we can notice that time is almost always related to more severe negative symptoms and patients who are educated, will have less severe negative symptoms. Table 2 analysis : patients who take their medications by force are the most compliant, although they represent a small number of the sample (n=3). Table number 3: there are no differences between either gender regarding the type of negative symptoms the patients develop.

**Disclosure of Interest:**

None Declared

## Long descriptions

### Table 1. Long description

The table consists of five columns and three rows. The header row lists the variables: Variables (spanning two columns), Age, Duration of the illness, and Educational level.

Row 1: Negative symptoms - Correlation Coefficient.

- Age: .303**

- Duration of the illness: 0.324**

- Educational level: -0.581**

Row 2: Negative symptoms - Sig. (2-tailed).

- Age: 0.005

- Duration of the illness: 0.002

- Educational level: 0.000

The double asterisks indicate statistical significance.

Navigate back to Table 1..

### Table 2. Long description

The first table is a Kruskal-Wallis test for differences in medication compliance and negative symptom severity. It contains five columns: Compliance to medications, N, Mean Rank, Kruskal-Wallis H, and Sig.

* Poor compliance: N equals 11, Mean Rank is 63.50.

* Moderate compliance: N equals 11, Mean Rank is 47.95.

* Good compliance: N equals 59, Mean Rank is 36.12.

* Forced compliance: N equals 3, Mean Rank is 71.00.

The Kruskal-Wallis H value for the group is 16.875 with a Sig. value of 0.000.

The second table is a T-test comparing gender. It contains seven columns: Gender, N dot, Mean, Std. Deviation, T, D f, and Sig.

* Male: N is 49, Mean is 26.65, Standard Deviation is 7.551.

* Female: N is 41, Mean is 23.93, Standard Deviation is 7.792.

The T value is 1.681, D f is 88, and Sig. is 0.096.

Navigate back to Table 2..

